# Gastrointestinal Carcinoma with Plasmacytoid Morphology: Positivity for c-MET, Arylsulfatase, and Markers of Epithelial-Mesenchymal Transition, as Indicators of Aggressivity

**DOI:** 10.1155/2019/5836821

**Published:** 2019-05-08

**Authors:** Zsolt Kovacs, Simona Gurzu, Calin Molnar, Mihaela Sincu, Laura Banias, Catalin Satala, Ioan Jung

**Affiliations:** ^1^Department of Pathology, University of Medicine, Pharmacy, Sciences and Technology, Tirgu-Mures, Romania; ^2^Department of Pathology, Clinical County Emergency Hospital, Tirgu-Mures, Romania; ^3^Department of Pathology, Research Center (CCAMF), Tirgu-Mures, Romania; ^4^Department of Surgery, University of Medicine, Pharmacy, Sciences and Technology, Tirgu-Mures, Romania

## Abstract

**Background:**

Plasmacytoid urothelial carcinoma is a rare and aggressive histologic variant of high-grade carcinoma of the urinary bladder. Few than 250 cases have been reported in the urinary bladder till January 2019. In this paper, a case series of unusual gastrointestinal carcinomas with plasmacytoid morphology was included. Only one similar case of the stomach was previously published and no such cases were found in colon.

**Methods:**

We present the complex immunoprofile, using a panel of 39 biomarkers, of the largest group of primary gastrointestinal carcinomas with plasmacytoid morphology reported in literature (one from upper rectum and six from stomach).

**Results:**

All of the seven cases showed lymph node metastases and only one survived over 25 weeks after surgical excision. The indicators of aggressivity were age (over 60), advanced stage (from IIIA to IV), E-cadherin negativity, and vimentin positivity. The immunoprofile indicated unfavorable prognosis for mesenchymal-type carcinomas (negativity for E-cadherin and positivity for vimentin, with membrane to nuclear translocation or negativity of *β*-catenin). The survivor showed an “epithelial-type adenocarcinoma with plasmacytoid dedifferentiation”, with membrane positivity for E-cadherin and *β*-catenin and vimentin negativity. All of the cases expressed c-MET and were negative for HER-2.

**Conclusions:**

Primary carcinoma with plasmacytoid morphology is a dedifferentiated variant of adenocarcinoma or poorly cohesive carcinomas. Vimentin positive dedifferentiated-poorly cohesive carcinomas should be considered as mesenchymal-type highly malignant carcinomas. This rare histologic variant of gastrointestinal cancer might respond to anti-c-MET tyrosine kinases.

## 1. Introduction

Plasmacytoid urothelial carcinoma (PUC) is a rare and aggressive histologic variant of high-grade carcinoma of the urinary bladder [1–4] whose diagnosis is difficult to make.

The PUC variant was described in the urinary bladder in 1991 [4] but was recognized by the World Health Organization (WHO) in 2004 [3, 5].

About 1-3% of all UCs are diagnosed as PUCs [5]. 61 cases have been reported in the English literature till 2012 [3], less than 100 cases till 2017 [3], and less than 250 till January 2019. They occur more frequently in males (M:F=3:1), around the age of 69 (range 46–87 years) [4, 6]. Due to their rare occurrence, few data are known about these carcinomas.

Although PUCs proved chemosensitive to cisplatin [4], they are usually diagnosed in late stages (pT3, pT4) [5], with metastases in 60% of the patients [5]. The median overall survival is 19-23 months (range: one week-43 months) [1–4, 6].

Except the urinary bladder, carcinomas with plasmacytoid morphology were also described involving other organs. Although CD138 may infrequently mark gastrointestinal carcinoma cells, only one case of primary gastric PC was reported in 2012 [7]. Another duodenal carcinoma with plasmacytoid morphology was reported in 2017 but finally proved to be metastatic tumors from a PUC and not a primary carcinoma [3]. WHO has not yet recognized this entity as histological subtype of gastric or colorectal carcinomas [8].

Independently from the localization, carcinoma with plasmacytoid morphology is characterized by diffuse proliferation of discohesive cells with plasmacytoid morphology, with eccentrically located nuclei, indistinct nucleoli, and eosinophilic cytoplasm that express cytokeratin (CK) and the transmembrane heparan sulfate proteoglycan CD138 (Syndecan-1) in over 50% of tumor cells [3–5]. The tumor cells can show solid sheet-like architecture or are arranged in cords and small nests [3].

In this paper, we present a comprehensive immunoprofile of 7 primary carcinomas with plasmacytoid morphology of the gastrointestinal tract: one from colon and 6 cases with gastric localization. The aim of the study was to identify the immunoprofile of the tumor cells, as a possible therapeutic target of this rare histologic variant of gastrointestinal carcinoma.

To have a complex immunohistochemical (IHC) picture of these tumors, we assessed the expression of a panel of 39 biomarkers that includes markers for diagnosis, epithelial-mesenchymal transition (EMT), adhesion molecules, markers of angiogenesis, and predictive markers. The obtained data were correlated with those obtained after a complex review of literature.

## 2. Material and Methods

### 2.1. Case Selection

We have retrospectively evaluated seven consecutive cases of primary gastrointestinal PCs (one of the colon and 6 of the stomach), diagnosed by our team in the last four years. No synchronous urothelial carcinoma or lobular carcinoma of the breast was associated with any of the included cases. No preoperative therapy was done.

The signed informed consent was obtained from all of the patients for publication of clinicopathological data.

**Figure 1:**
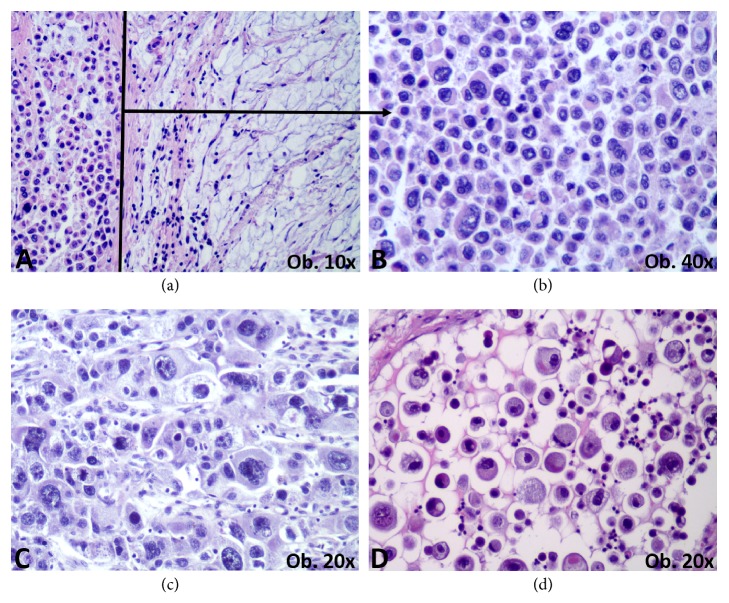
Representative aspect of carcinoma with plasmacytoid morphology, with discohesive round to ovoid cells with eccentrically located nuclei (a-left, b–d), sometimes with nuclear pleomorphism (c). The mucinous adenocarcinoma can be associated (a-right).

We reviewed the Hematoxylin and Eosin- (HE-) stained slides to confirm diagnosis and quantify the percentage and microscopic subtype of adenocarcinoma versus carcinoma with plasmacytoid morphology component. All cases presented at least 80% plasmacytoid component (Table 1). It was evaluated based on the presence of round to ovoid discohesive cells, with eccentrically located nuclei (Figure 1).

The 8th edition of AJCC staging system [8] was used for establishing the pTNM stage. The Dukes-MAC stage was also appreciated based on new literature proposal [9].

### 2.2. Immunohistochemistry

**Figure 2:**
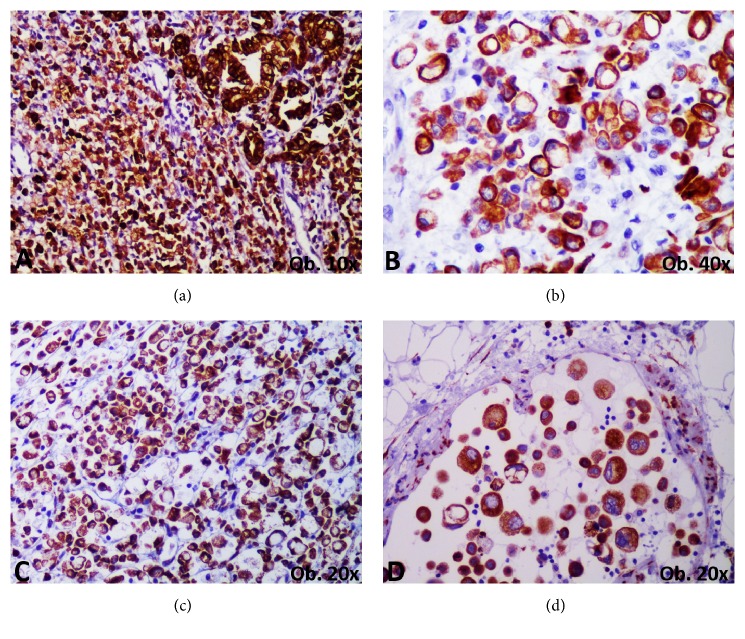
The diagnosis of primary gastrointestinal carcinoma with plasmacytoid morphology is based on positivity for cytokeratin 20 (a) or cytokeratin 7 (b) and simultaneous positivity for CD138 (c, d).

In all of the cases, a complex immunoprofile of the tumor cells was done to perform differential diagnosis of a primary versus metastatic tumor (Table 2). The diagnosis of carcinoma with plasmacytoid morphology was suspected in HE and confirmed by double positivity for CD138 and cytokeratins (CK AE1/AE3 and CK7 or CK20) (Table 3 and Figure 2). As we have mentioned before, at least 80% plasmacytoid component was identified in all of the cases. CD138 marked the plasmacytoid component only, without positivity in the adenocarcinoma/poorly cohesive carcinoma (including signet ring cells) component. No stromal positivity was noted.

**Figure 3:**
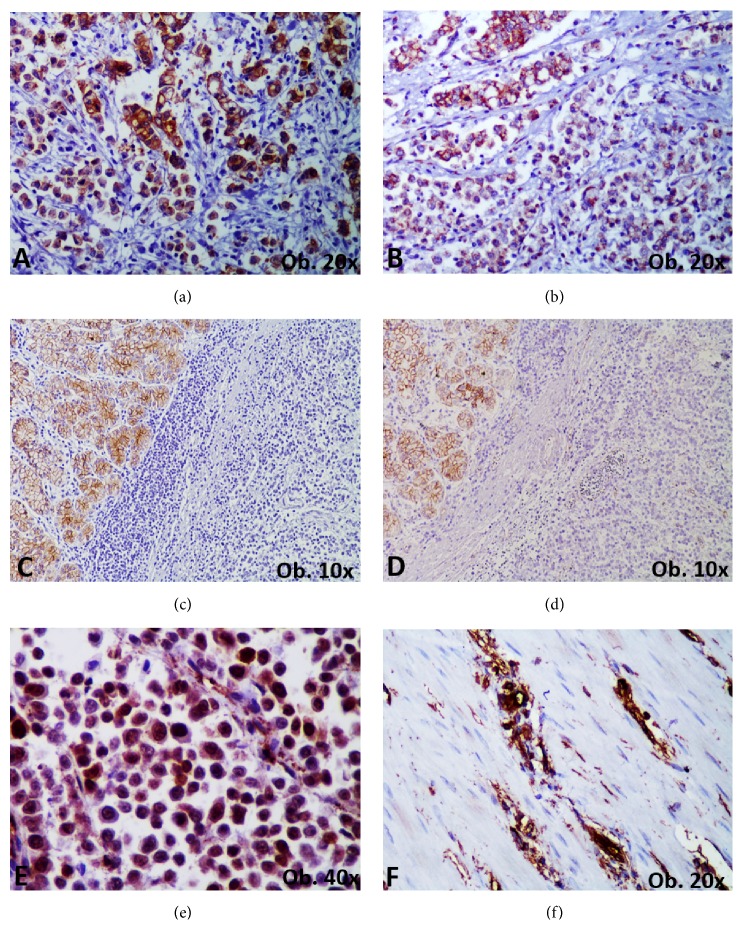
Epithelial-mesenchymal transition (EMT) of gastrointestinal carcinoma with plasmacytoid morphology. The “epithelial-type carcinoma with plasmacytoid morphology” is characterized by membrane positivity for E-cadherin (a) and *β*-catenin (b). In the “mesenchymal-type carcinoma with plasmacytoid morphology”, loss of E-cadherin (c-right) and *β*-catenin (d-right) or nuclear translocation of *β*-catenin (e) can be seen. Vimentin positivity (f) is also characteristics of carcinomas with EMT transition.

The EMT was analyzed using the IHC markers of the Wnt pathway E-cadherin, *β*-catenin, N-cadherin, vimentin, and arylsulfatase A and B (ARSA, ARSB). CD44 was used to explore the stemness features of the tumor cells. Those cases showing loss of E-cadherin with membrane to nuclear translocation of *β*-catenin, or negativity for *β*-catenin, were considered as showing EMT. Positivity for N-cadherin and vimentin was also checked for identification of mesenchymal features. As the adhesion molecule V-set and immunoglobulin (VSIG) and SLUG were positive in all of the cases and N-cadherin and smooth muscle actin (SMA) was negative, the tumors were classified as epithelial-type carcinomas (positive for E-cadherin, with membrane expression of *β*-catenin and negativity for vimentin) or mesenchymal-type carcinomas (negative for E-cadherin, with nuclear expression of *β*-catenin or positivity for vimentin). The other cases were considered as having a hybrid EMT phenotype (Table 3 and Figure 3).

## 3. Results

### 3.1. Clinicopathological Data

In our university hospital, there are about 60 gastric carcinomas and 150 colorectal carcinomas diagnosed every year. The 7 primary carcinomas with plasmacytoid morphology (one from upper rectum and 6 from stomach) represented about 0.16% of all colorectal carcinomas and 2.5% of all gastric carcinomas diagnosed in our department of pathology during 2016-2019. They were identified in patients with a median age of 70.43±11.24 years (range: 52 to 83 years) and a report of M:F=2.5:1.

All of the patients were diagnosed in metastatic stages, with invasion in lymphatic (L1) and/or blood vessels (V1) and extremely short overall survival (Table 1). All of the tumors were removed with free resection margins (R0). Only one patient (the youngest one: 52 years old) is alive at 25 weeks after surgery (case 3). As the tumor cells did not express HER-2, classic chemotherapy was administrated. The other 6 patients died between 3 and 23 weeks (below six months) after surgery (Table 1).

### 3.2. Histological Diagnosis

The diagnosis of a primary carcinoma was histologically based on the origin of tumor cells within gastric or colorectal mucosa.

In carcinoma with plasmacytoid morphology of the upper rectum (Case 1, Table 1), it was about a mucinous adenocarcinoma with signet ring cell component, with plasma cells-like discohesive cells, in the invasion front (Figure 1). As the surgical intervention was made in emergency, due to mechanical ileus, no preoperative chemoradiotherapy was done.

All of the six gastric carcinomas were of poorly cohesive-type, with/without signet ring cell component, with 10% component of adenocarcinoma, in cases 2 and 6 (Table 1).

### 3.3. Immunohistochemistry

#### 3.3.1. Primary versus Metastatic Tumor

The colonic origin was proved by positivity of tumor cells for CK20 and inconstant positivity for CDX2. Gastric origin was revealed by inconstant positivity for CK7 and/or CK20 (Figure 2). Lymphoma was excluded based on negativity for Leukocyte Common Antigen (LCA), CD20, and CD3, and three neuroendocrine markers (chromogranin, synaptophysin, neuron specific enolase [NSE]) were used to exclude a neuroendocrine carcinoma (Table 3).

Metastases from a PUC were excluded based on positivity for CK20 and/or CDX2 and carcinoembryonic antigen (CEA) and negativity for GATA3. Metastases from a lobular carcinoma of the breast were based on negativity for Estrogen and Progesteron receptors (ER, PgR) and also negativity for mammaglobin, endothelial transcription factor 3 (GATA 3), and gross cystic disease fluid protein 15 (GCDFP-15). S100 and HMB45 negativity excluded a metastatic melanoma (Tables 3 and 4).

#### 3.3.2. Microsatellite Status

The microsatellite status was IHC assessed using the markers MLH-1, MSH-2, PMS-2, and MSH-6. As all of the markers were positive (Table 3), all tumors were considered proficient for mismatch repair proteins (MMR-proficient), respectively, microsatellite stable (MSS) carcinomas.

#### 3.3.3. Epithelial-Mesenchymal Transition

From 6 gastric carcinomas with plasmacytoid morphology, 4 cases were classified as mesenchymal-type carcinomas, one as epithelial type, and one as having a hybrid phenotype (positivity for VSIG and SLUG only), the same as the mesenchymal-type carcinoma with plasmacytoid morphology of the upper rectum (Table 3). The longer survival (over 25 weeks) was seen for the epithelial-type carcinoma (case 2) (Tables 1 and 3 and Figure 3).

#### 3.3.4. Angiogenesis

All of the cases showed negativity for Vascular Endothelial Growth Factor A (VEGF-A), as expression of lack of angiogenic immunophenotype. Negativity for maspin was an indicator of high risk for distant metastases.

#### 3.3.5. Predictive Markers

**Figure 4:**
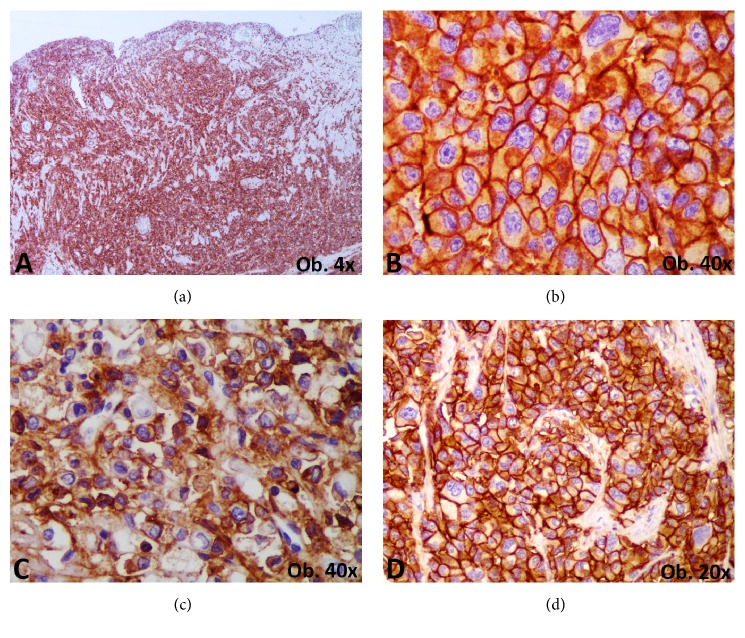
Predictive markers of carcinoma with plasmacytoid morphology c-MET (a, b) and CD44 (c, d) are expressed in the tumor cells cytoplasm (a, c) or membrane (b, d).

No positivity for HER-2, c-KIT, NGAL, or CD10 was observed but all of the cases diffusely expressed c-MET protein (Figure 4).

## 4. Discussion

To differentiate a primary carcinoma with plasmacytoid morphology of gastrointestinal tract from a metastatic tumor, especially from PUC or breast lobular carcinoma, a complex immunoprofile is necessary (Table 4). Primary lymphomas, as plasma cell lymphoma, and carcinoma variants should also be excluded [3].

Although carcinoma with plasmacytoid morphology expresses CD138, the diagnosis is based on simultaneous positivity for pan-cytokeratin (CK AE1/AE3) and epithelial membrane antigen (EMA) [3, 5]. In contrast with lymphomas, Leukocyte Antigen (LCA), multiple myeloma 1/interferon regulatory factor 4, and k and l light chains are negative in PCs [4, 5].

CK AE1/AE3 marks 97% of carcinomas with plasmacytoid morphology [3], independently of their localization. Regarding the CK variants, CK20 is usually positive for colorectal carcinomas but gastric carcinomas with plasmacytoid morphology and PUCs can also express this marker. The gastrointestinal versus urothelial origin cannot be based on keratin 7. It is positive for urothelial carcinoma but can also mark the gastric and colorectal carcinomas, especially those with microsatellite status or with serrated pathway and BRAF mutations [7, 10].

CDX2 and polyclonal carcinoembryonic antigen (p-CEA) may mark both colorectal carcinomas with plasmacytoid morphology and PUCs but uroplakin is positive for PUCs only [1, 4]. CDX2 is rarely positive in gastric carcinomas, as in our cases.

The suspicion of a metastasis from a lobular carcinoma of the breast is eliminated based on negativity for specific markers such mammaglobin, ER, PR, and GATA 3 [1, 3, 11, 12]. However, the poorly cohesive gastric carcinomas can express ER [1, 11, 12] and urothelial carcinoma can be diffusely positive for GCDFP-15 [1] and express nuclear GATA3 [1, 3]. CD138 can mark the breast lobular carcinoma but its expression is simultaneously seen in tumor and stroma cells [13]. E-cadherin is negative in over 75% of invasive lobular carcinoma of the breast, as a result of mutations of the* CDH1* gene [13, 14]. Loss of E-cadherin occurs in parallel with decreased *β*-catenin expression [14]. GCDFP-15 is commonly negative in invasive breast lobular carcinoma. Whereas HER-2, ER and PR are used as predictive factors,* c-MET* aberrations (mutations or amplification) are indicators of high-grade invasive breast lobular carcinomas with increased metastatic risk and are commonly identified in triple negative basal-like cases [15] that represent below 2% of all invasive lobular carcinomas of the breast [16].

Similar to our study, it was shown that E-cadherin is mostly negative in PUC, as marker of aggressivity and activated Wnt pathway [2, 5, 6] but vimentin can be positive or negative [4]. S100, a marker of EMT, was also found negative in the reported PUCs [6], as in our cases.

PUC shows a predilection for intraperitoneal spread and carcinomatous ascites [3, 4]. As CA-125, the marker usually used for diagnosis of ovarian cancer, can rise in the serum of patients [3], the differential diagnosis of metastatic carcinoma with plasmacytoid morphology is extremely difficult in females. Similar to colorectal carcinomas, serum CEA can also be high in patients with PUC [4]. CA19-9 and *β*-HCG were also reported to be increased [6]. For any patient with peritoneal carcinomatosis, the primary tumor should be checked in ovary, gastrointestinal tract, and urinary bladder.

As we have mentioned in the Introduction, only one case was reported in literature as gastric carcinoma with plasmacytoid morphology, in a 66-year-old male [4]. Differentiation between a poorly cohesive gastric carcinoma and the carcinoma with plasmacytoid morphology variant is based on CD138 positivity in over 50% of tumor cells [3]. In the present material, the median age of patients was 70.43±11.24 years (range: 52 to 83 years), which is significantly higher than that in other gastric carcinomas previously reported in our department: 62.19±13.96 (range 21–98 years) [9]. The carcinoma with plasmacytoid morphology of the stomach presented, in our material, several negative prognostic factors [8, 9, 15]: age over 50, advanced stage (both pTNM and Dukes-MAC like), angiolymphatic invasion, EMT phenotype, and positivity for c-MET and CD44. CD44 is a cancer stem marker that seems to induce chemoresistance [12].

As CD138 is an extracellular matrix receptor involved in intercellular communication, proliferation, angiogenesis, and metastasis [3], we consider that it should be considered as an indicator of poorly cohesive carcinoma aggressivity, independent of the tumor location. It probably interacts with the Wnt and ARSA/ARSB pathways and is involved in the process of EMT of carcinoma cells.

Our case series showed that, in gastrointestinal tract, carcinoma with plasmacytoid morphology is an aggressive “mesenchymal-type poorly cohesive carcinoma” that expresses c-MET but not HER-2. This immunophenotype indicates a possibility of the response of these tumors to tyrosine kinase inhibitors that target MET signaling, such as imatinib or foretinib, which are currently used in clinical trials, in patients with solid tumors. This aspect should be proved in large cohorts.

## Figures and Tables

**Table 1:** The clinicopathological features of patients with gastrointestinal carcinomas with plasmacytoid morphology (∗ means upon 9, and ∗∗ means 8).

Case no	Gender	Age (years)	Tumor localization	Macroscopy	Microscopy – adenocarcinoma type and part (%)	Microscopy – plasmacytoid part (%)	pT stage	pN stage	Angio-lymphatic invasion	pM stage	Dukes-MAC (Dukes-MAC like∗) stage	WHO's stage∗∗	OS

1	M	62	Upper rectum	Ulceroinfiltrative	10% - mucinous adenocarcinoma and 10% - signet ring cells component	80%	4a	N2 (13/13)	L1V1	M0	D	IIIC	Died at 3 weeks

2	M	52	Small curvature – proximal stomach	Ulceroinfiltrative	10% G3 adenocarcinoma and 10% poorly cohesive carcinoma	80%	4a	N2 (4/30)	L1V0	M0	D	IIIA	Alive at 25 weeks

3	M	70	Small curvature – Distal stomach	Polypoid-ulcerated	10% - Poorly cohesive carcinoma	90%	4b	N3b (19/22)	L1V0	M0	D	IIIC	Died at 20 weeks

4	M	67	Greater curvature – Proximal and distal stomach	Linitis plastica (Infiltrative)	10% - Poorly cohesive carcinoma	90%	4b	N3a (14/55)	L1V1	M0	D	IIIC	Died at 23 weeks

5	F	83	Small curvature – Proximal and distal stomach	Linitis plastica (Infiltrative)	10% - Signet ring cell carcinoma	90%	4a	N3a (13/46)	L1V1	M1 (abdominal carcinomatosis)	D	IV	Died at 4 weeks

6	M	82	Antrum	Ulceroinfiltrative	10% G2 adenocarcinoma and 10% poorly cohesive carcinoma	80%	2	N3b (25/26)	L1V1	M0	B2	IIIB	Died at 4 weeks

7	F	77	Small curvature – proximal stomach	Ulceroinfiltrative	10% - Poorly cohesive carcinoma	90%	3	N3b (18/39)	L1V1	M1 (metastases in pericaval lymph nodes)	D	IV	Died at 15 weeks

**Table 2:** The immunohistochemical markers used in the study.

Marker	Supplier	Clone	Dilution	Scoring
CD138	Dako, Glostrup, Denmark	MI 15	RTU	≥50% cytoplasmic or membrane staining
CDX2	Dako	DAK-CDX2	RTU	≥1% nuclear staining
CEA	Thermo Scientific, San Diego, CA, USA	Ab3	1:200	≥1% cytoplasmic staining
CK AE1/AE3	Dako	AE1/AE3	1:100	≥1% cytoplasmic or membrane staining
CK20	Thermo Scientific	Q6	1:100	≥1% cytoplasmic or membrane staining
CK7	Thermo Scientific	OV-TL 12/30	1:100	≥1% cytoplasmic or membrane staining
MLH-1	Leica	ESO5	1:50	≥1% nuclear staining
MSH-2	Leica	25D12	1:50	≥1% nuclear staining
PMS-2	Leica	Monoclonal	1:50	≥1% nuclear staining
MSH-6	Leica	Monoclonal	1:50	≥1% nuclear staining
SLUG	Santa Cruz	Polyclonal	1:100	Cytoplasmic positivity – 1% cut-off
E-cadherin	Dako	NCH-38	1:50	Membrane positivity – 5% cut-off
*β*-catenin	Leica	17 C2	1:50	Membrane, cytoplasmic or nuclear positivity – 5% cut-off
N-cadherin	Dako	6G11	1:100	Membrane or cytoplasmic positivity – 1% cut-off
Vimentin	Dako	V9	1:800	Cytoplasmic positivity – 10% cut-off
c-met	Abcam, Cambridge, UK	Monoclonal	1:2000	Cytoplasmic positivity – 5% cut-off
VSIG	Sigma Aldrich	Polyclonal	1:2500	Cytoplasmic or membrane positivity – 5% cut-off
S100	Thermo Scientific	Polyclonal	1:8000	Cytoplasmic positivity – 5% cut-off
SMA	Dako	1A4	RTU	Cytoplasmic positivity – 1% cut-off
CD44	Leica	DF1485	1:50	Cytoplasmic or membrane positivity – 10% cut-off
Maspin	Santa Cruz	H-130	1:25	Cytoplasmic or nuclear positivity – 5% cut-off
VEGF-A	Novocastra	VG1	1:50	Cytoplasmic positivity – 5% cut-off
Synaptophysin	Dako	DAK-SYNAP	RTU	Cytoplasmic or membrane positivity – 5% cut-off
Chromogranin	Dako	DAK A3	RTU	Cytoplasmic or membrane positivity – 5% cut-off
NSE	Dako	M0873	1:100	Cytoplasmic or membrane positivity – 5% cut-off
ER	Dako	1D5	RTU	Nuclear positivity - 1% cut-off
PR	Dako	PgR636	RTU	Nuclear positivity - 1% cut-off
HER-2	Dako	Polyclonal	1:200	Membrane positivity – HercepTestTM guidelines
Melan A	Dako	A103	RTU	Cytoplasmic positivity – 1% cut-off
HMB45	Cell Marque	Monoclonal	RTU	Cytoplasmic positivity – 1% cut-off

**Table 3:** Immunohistochemical profile of gastrointestinal carcinoma with plasmacytoid morphology included in the study.

Biomarker group	Biomarker name	Case number						
		Case 1	Case 2	Case 3	Case 4	Case 5	Case 6	Case 7
***Plasmacytoid differentiation***	*CD138*	Positive	Positive	Positive	Positive	Positive	Positive	Positive
	*CD38*	Focal positive	Focal positive	Negative	Focal positive	Positive	Positive	Positive

***Epithelial origin***	*CK AE1/AE3*	Positive	Positive	Positive	Positive	Positive	Positive	Positive
	*CK7*	Negative	Positive	Negative	Positive	Positive	Positive	Positive
	*CK20*	Positive	Negative	Negative	Negative	Negative	Negative	Negative
	*CDX2*	Positive	Negative	Negative	Negative	Negative	Negative	Negative
	*CEA*	Positive	Negative	Negative	Negative	Negative	Negative	Negative

***Microsatellite status***	*MLH-1*	Positive	Positive	Positive	Positive	Positive	Positive	Positive
	*MSH-2*	Positive	Positive	Positive	Positive	Positive	Positive	Positive
	*PMS-2*	Positive	Positive	Positive	Positive	Positive	Positive	Positive
	*MSH-6*	Positive	Positive	Positive	Positive	Positive	Positive	Positive

***Epithelial-mesenchymal transition (EMT)***	*SLUG*	Positive	Positive	Positive	Positive	Positive	Positive	Positive
	*E-cadherin*	Negative	Positive	Negative	Negative	Negative	Negative	Negative
	*N-cadherin*	Negative	Negative	Negative	Negative	Negative	Negative	Negative
	*β-catenin*	Positive - Nuclear	Positive - membrane	Negative	Negative	Positive -membrane	Negative	Negative
	*Vimentin*	Negative	Negative	Positive	Positive	Positive	Negative	Negative
	*VSIG*	Focal positive	Positive	Positive - cytoplasm	Positive	Positive - membrane	Positive - cytoplasm	Positive
	*CD44*	Positive >25%	Negative	Positive >50%	Positive >50%	Positive >50%	Negative	Negative
	*Arylsulfatase A*	Positive	Positive	Positive	Positive	Positive	Positive	Negative
	*Arylsulfatase B*	Positive	Positive	Positive	Negative	Positive	Positive	Positive
	*SMA*	Negative	Negative	Negative	Negative	Negative	Negative	Negative
	***EMT subtype***	***Mesenchymal***	***Epithelial***	***Mesenchymal***	***Mesenchymal***	***Mesenchymal***	***Hybrid***	***Mesenchymal***

***Angiogenesis***	*Maspin*	Negative	Negative	Negative	Negative	Negative	Negative	Nuclear

	*VEGF-A*	Negative	Negative	Negative	Negative	Negative	Negative	Negative

***Differential diagnosis***								
*Neuroendocrine markers*	*Chromogranin, NSE, synaptophysin*	Negative	Negative	Negative	Negative	Negative	Negative	Negative
*Lymphoid differentiation*	*CD20, CD3, LCA, CD15*	Negative	Negative	Negative	Negative	Negative	Negative	Negative
*Breast origin*	*Mammaglobin, GATA3*	Negative	Negative	Negative	Negative	Negative	Negative	Negative
*Melanoma origin*	*S100, HMB45*	Negative	Negative	Negative	Negative	Negative	Negative	Negative

***Predictive markers***	*c-MET*	Positive	Positive	Positive	Positive	Positive	Positive	Positive
	*HER-2*	Negative	Negative	Negative	Negative	Negative	Negative	Negative
	*C-KIT*	Negative	Negative	Negative	Negative	Negative	Negative	Negative
	*NGAL*	Negative	Negative	Negative	Negative	Negative	Negative	Negative
	*CD10*	Negative	Negative	Negative	Negative	Negative	Negative	Negative

**Table 4:** The immunoprofile of primary carcinoma with plasmacytoid morphology versus metastatic tumors in the gastrointestinal tract (adapted upon 1, 2, 3, 5, 7, 12-16).

BIOMARKER	Gastric PC	Colorectal PC	Urothelial PC	Invasive lobular carcinoma of the breast
***CD138***	100% positivity	100% positivity	~78% positivity	~90% positivity in metastatic cases, in epithelium and/or stroma

***CK AE1/AE3***	97% positivity	100% positivity	~97% positivity	Usually positive

***CK7***	Usually positive	Sporadic positive	~77.4% positivity	Usually positive

***CK20***	Negative or focally positive	~97% positivity	~72% focal positivity	Usually negative

***CDX2***	Usually negative	Usually positive	~18% positivity	Negative

***CEA***	Usually negative	Positive	~49% positivity	Negative

***Uroplakin II***	Negative	Negative	~33% positivity	Negative

***ER***	Usually negative	Usually negative	Negative	~95% positivity

***PR***	Negative	Negative	~13% positivity	~76-83% positivity

***Mammaglobin***	Negative	Negative	Negative	Usually positive

***GCPDFP-15***	Negative	Negative	~24% positivity	Usually negative

***GATA 3***	Negative or weak and sporadic	Negative	~70-88% positivity	≥90% positivity (only 22% in triple negative tumors)

***MLH-1***	Usually positive, usually MSS	Usually positive, usually MSS	No data in PubMed cited papers	Usually positive, usually MSS

***MSH-2***	Usually positive	Usually positive	No data in PubMed cited papers	Usually positive

***PMS-2***	Usually positive	Usually positive	No data in PubMed cited papers	No data in PubMed cited papers

***MSH-6***	Usually positive	Usually positive	No data in PubMed cited papers	No data in PubMed cited papers

***E-cadherin***	Usually negative	Usually negative	~25% positivity, 75% diminished or negative	~10-25% membrane or aberrant nuclear positivity

***β-catenin***	Negative or membrane positivity	Nuclear positivity	22.5% negative, 17% nuclear positivity, 60.5% membrane positivity	~90% reduction or complete loss of positivity, 8-10% membrane or cytoplasmic positivity

***Vimentin***	Usually positive	Negative	Usually negative	~14% positivity

***N-cadherin***	Usually negative	Usually negative	No data in PubMed cited papers	~4% positivity

***VSIG1***	Usually positive	Usually positive	No data in PubMed cited papers	Usually negative

***SLUG***	Usually positive	Usually positive	No data in PubMed cited papers	~2-4% positivity

***CD44***	Positive or negative	Positive or negative	No data in PubMed cited papers	Positive in metastatic or multidrug resistant cases

***Maspin***	Negative	Negative or nuclear positivity	No data in PubMed cited papers	~7% cytoplasmic positivity

***S100***	Negative	Negative	Negative	~57% positivity

***VEGF-A***	Negative	Negative	No data in PubMed cited papers	Negative or positive

***HER-2***	Negative	Negative		Usually negative

***c-MET***	Usually positive	Usually positive	No data in PubMed cited papers	Positive in triple negative metastatic cases

## Data Availability

The clinicopathological data used to support the findings of this study are available from the corresponding author upon request.
